# 2-(3-Oxo-2,3-dihydro-1,2-benzothia­zol-2-yl)acetic acid

**DOI:** 10.1107/S1600536811047490

**Published:** 2011-11-12

**Authors:** Xiang-hui Wang, Jian-Xin Yang, Cheng-hang You, Xue-mei Tan, Qiang Lin

**Affiliations:** a Institute of Environmental Science and Engineering, Kunming University of Science and Technology, Kunming 650093, People’s Republic of China; b College of Materials and Chemical Engineering, Hainan University, Haikou 570228, People’s Republic of China; c College of Chemistry and Chemical Engineering, Hainan Normal University, Haikou 571100, People’s Republic of China

## Abstract

In the title compound, C_9_H_7_NO_3_S, the benzoisothia­zolone ring system is essentially planar, with a maximum deviation of 0.013 (2) Å. In the crystal, mol­ecules are linked *via* O—H⋯O hydrogen bonds, forming chains along [010]. In addition, weak inter­molecular C—H⋯O hydrogen bonds are present.

## Related literature

For background to the sythesis of benzisothia­zolone derivatives, see: Davis (1972 ▶); Maggiali *et al.* (1982 ▶, 1983 ▶), Elgazwy & Abdel-Sattar (2003 ▶). For details of their biological activity, see: Taubert *et al.* (2002 ▶); Mor *et al.* (1996 ▶). For related structures, see: Xu *et al.* (2006 ▶), Wang *et al.* (2011*a* ▶,*b* ▶,*c* ▶).
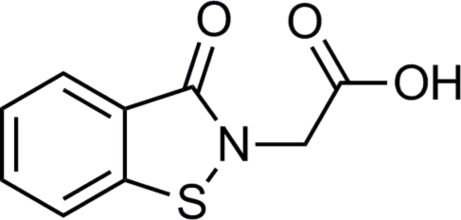


## Experimental

### 

#### Crystal data

C_9_H_7_NO_3_S
*M* *_r_* = 209.22
Orthorhombic, 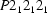

*a* = 4.7774 (11) Å
*b* = 11.367 (3) Å
*c* = 16.159 (4) Å
*V* = 877.6 (4) Å^3^
*Z* = 4
Mo *K*α radiation
μ = 0.35 mm^−1^
*T* = 153 K
0.29 × 0.22 × 0.20 mm

#### Data collection

Rigaku AFC10/Saturn724+ diffractometer
Absorption correction: multi-scan (*ABSCOR*; Higashi, 1995 ▶) *T*_min_ = 0.907, *T*_max_ = 0.934
7675 measured reflections
2340 independent reflections
2141 reflections with *I* > 2σ(*I*)
*R*_int_ = 0.035

#### Refinement

*R*[*F* ^2^ > 2σ(*F* ^2^)] = 0.032
*wR*(*F* ^2^) = 0.068
*S* = 1.00
2340 reflections
131 parameters
H atoms treated by a mixture of independent and constrained refinement
Δρ_max_ = 0.27 e Å^−3^
Δρ_min_ = −0.22 e Å^−3^
Absolute structure: Flack (1983 ▶), 945 Friedel pairs
Flack parameter: 0.08 (7)

### 

Data collection: *CrystalClear* (Rigaku, 2008 ▶); cell refinement: *CrystalClear*; data reduction: *CrystalClear*; program(s) used to solve structure: *SHELXS97* (Sheldrick, 2008 ▶); program(s) used to refine structure: *SHELXL97* (Sheldrick, 2008 ▶); molecular graphics: *SHELXTL* (Sheldrick, 2008 ▶) and *DIAMOND* (Brandenburg, 1999 ▶); software used to prepare material for publication: *SHELXTL* and *publCIF* (Westrip, 2010 ▶).

## Supplementary Material

Crystal structure: contains datablock(s) I, global. DOI: 10.1107/S1600536811047490/lh5367sup1.cif

Structure factors: contains datablock(s) I. DOI: 10.1107/S1600536811047490/lh5367Isup2.hkl

Supplementary material file. DOI: 10.1107/S1600536811047490/lh5367Isup3.cml

Additional supplementary materials:  crystallographic information; 3D view; checkCIF report

## Figures and Tables

**Table 1:** Hydrogen-bond geometry (Å, °)

*D*—H⋯*A*	*D*—H	H⋯*A*	*D*⋯*A*	*D*—H⋯*A*
O3—H3*O*⋯O1^i^	0.86 (3)	1.72 (3)	2.581 (2)	173 (3)
C2—H2⋯O2^ii^	0.95	2.60	3.310 (2)	132
C8—H8*A*⋯O2^iii^	0.99	2.34	3.246 (2)	152

Symmetry codes: (i) 
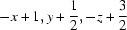
; (ii) 
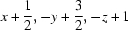
; (iii) 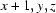.

**Fig. 1.**
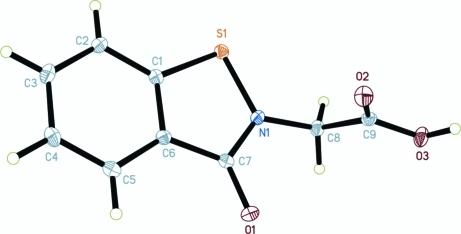
The molecular structure of the title compound with displacement ellipsoids drawn at the 30% probability level. H atoms are presented as a small spheres of arbitrary radius.

**Fig. 2.**
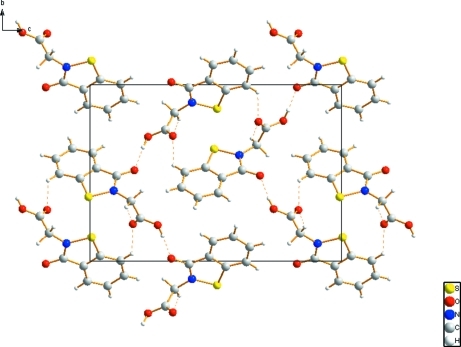
Part of the crystal structure with hydrogen bonds drawn as dashed lines.

## supplementary crystallographic information

### Comment

2-(3-Oxobenzo[*d*]isothiazol-2(3*H*)-yl)acetic acid is an important intermediate in the synthesis of benzisothiazolone derivatives (Davis, 1972; Maggiali, *et al.*, 1982,1983; Elgazwy & Abdel-Sattar, 2003). The corresponding esters and amides have been reported to possess high antibacterial and antifungal activity (Mor *et al.*, 1996; Taubert *et al.*, 2002). In view of the importance of 1,2-benzisothiazol-3(2*H*)-ones, the title compound, (I), was synthesized and its crystal structure is presented herein.

The molecular structure of the title compound (I) is shown in Fig. 1. Examples of related structures appear in the literature (Xu, *et al.*, 2006; Wang, *et al.*, 2011*a*,*b*,*c*). In (I) the benzoisothiazolone ring system is essentially planar, with a maximum deviation of 0.013 (2) Å. In the crystal, molecules are linked *via* O—H···O hydrogen bonds to form one-dimensional chains along [010]. In addition weak intermolecular C—H···O hydrogen bonds are present.

### Experimental

Chloroactic acid (0.95 g, 0.01 mol) was added dropwise to a solution of sodium hydroxide (0.80 g, 0.02 mol) and benzo[*d*]isothiazol-3(2*H*)-one (1.50 g, 0.01 mol)in water (20 ml) under stirring on an ice-water bath. The reaction mixture was stirred at room temperature for 4.5 h and adjusted pH to 1~2, to afford the title compound (1.05 g, yield 50.0%). Single crystals suitable for X-ray measurements were obtained by recrystallization of the title compound from the mixed solution of dimethyl formamide and water at room temperature.

### Refinement

Atom H3O was located from the difference Fourier map and was refined freely [O–H = 0.86 (3) Å]. The remaining H atoms bonded to C atoms were fixed geometrically and allowed to ride on their attached atoms, with the carrier atom-H distances = 0.95 Å for aryl, 0.99 for methylene, and *U*_iso_(H) = 1.2U_eq_(C).

### Crystal data

**Table:**

C_9_H_7_NO_3_S	*F*(000) = 432
*M**_r_* = 209.22	*D*_x_ = 1.584 Mg m^−^^3^
Orthorhombic, *P*2_1_2_1_2_1_	Mo *K*α radiation, λ = 0.71073 Å
Hall symbol: P 2ac 2ab	Cell parameters from 3259 reflections
*a* = 4.7774 (11) Å	θ = 3.1–29.1°
*b* = 11.367 (3) Å	µ = 0.35 mm^−^^1^
*c* = 16.159 (4) Å	*T* = 153 K
*V* = 877.6 (4) Å^3^	Block, colorless
*Z* = 4	0.29 × 0.22 × 0.20 mm

### Data collection

**Table:**

Rigaku AFC10/Saturn724+ diffractometer	2340 independent reflections
Radiation source: Rotating Anode	2141 reflections with *I* > 2σ(*I*)
graphite	*R*_int_ = 0.035
Detector resolution: 28.5714 pixels mm^-1^	θ_max_ = 29.1°, θ_min_ = 3.1°
φ and ω scans	*h* = −6→6
Absorption correction: multi-scan (*ABSCOR*; Higashi, 1995)	*k* = −15→15
*T*_min_ = 0.907, *T*_max_ = 0.934	*l* = −22→16
7675 measured reflections	

### Refinement

**Table:**

Refinement on *F*^2^	Secondary atom site location: difference Fourier map
Least-squares matrix: full	Hydrogen site location: inferred from neighbouring sites
*R*[*F*^2^ > 2σ(*F*^2^)] = 0.032	H atoms treated by a mixture of independent and constrained refinement
*wR*(*F*^2^) = 0.068	*w* = 1/[σ^2^(*F*_o_^2^) + (0.0304*P*)^2^ + 0.136*P*] where *P* = (*F*_o_^2^ + 2*F*_c_^2^)/3
*S* = 1.00	(Δ/σ)_max_ < 0.001
2340 reflections	Δρ_max_ = 0.27 e Å^−^^3^
131 parameters	Δρ_min_ = −0.22 e Å^−^^3^
0 restraints	Absolute structure: Flack (1983), 945 Friedel pairs
Primary atom site location: structure-invariant direct methods	Flack parameter: 0.08 (7)

### Special details

**Table:**

Geometry. All e.s.d.'s (except the e.s.d. in the dihedral angle between two l.s. planes) are estimated using the full covariance matrix. The cell e.s.d.'s are taken into account individually in the estimation of e.s.d.'s in distances, angles and torsion angles; correlations between e.s.d.'s in cell parameters are only used when they are defined by crystal symmetry. An approximate (isotropic) treatment of cell e.s.d.'s is used for estimating e.s.d.'s involving l.s. planes.
Refinement. Refinement of *F*^2^ against ALL reflections. The weighted *R*-factor *wR* and goodness of fit *S* are based on *F*^2^, conventional *R*-factors *R* are based on *F*, with *F* set to zero for negative *F*^2^. The threshold expression of *F*^2^ > σ(*F*^2^) is used only for calculating *R*-factors(gt) *etc*. and is not relevant to the choice of reflections for refinement. *R*-factors based on *F*^2^ are statistically about twice as large as those based on *F*, and *R*- factors based on ALL data will be even larger.

### Fractional atomic coordinates and isotropic or equivalent isotropic displacement parameters (Å^2^)

**Table:**

	*x*	*y*	*z*	*U*_iso_*/*U*_eq_	
S1	0.79701 (8)	0.63585 (3)	0.49404 (2)	0.01821 (10)	
O1	0.4484 (3)	0.48499 (11)	0.67636 (7)	0.0275 (3)	
O2	0.4545 (3)	0.79003 (11)	0.67052 (8)	0.0253 (3)	
O3	0.7852 (3)	0.79542 (12)	0.76921 (7)	0.0266 (3)	
N1	0.7188 (3)	0.60058 (12)	0.59414 (8)	0.0196 (3)	
C1	0.5586 (3)	0.53095 (14)	0.45995 (10)	0.0170 (3)	
C2	0.4940 (4)	0.50367 (15)	0.37799 (10)	0.0208 (4)	
H2	0.5850	0.5422	0.3333	0.025*	
C3	0.2934 (4)	0.41885 (16)	0.36417 (11)	0.0251 (4)	
H3	0.2443	0.3992	0.3089	0.030*	
C4	0.1598 (4)	0.36082 (17)	0.42984 (11)	0.0251 (4)	
H4	0.0224	0.3027	0.4184	0.030*	
C5	0.2255 (3)	0.38708 (13)	0.51040 (11)	0.0216 (3)	
H5	0.1357	0.3475	0.5548	0.026*	
C6	0.4278 (4)	0.47343 (14)	0.52566 (10)	0.0176 (3)	
C7	0.5239 (4)	0.51590 (14)	0.60561 (10)	0.0191 (4)	
C8	0.8509 (4)	0.65927 (15)	0.66335 (10)	0.0212 (4)	
H8A	1.0303	0.6939	0.6447	0.025*	
H8B	0.8936	0.6005	0.7067	0.025*	
C9	0.6707 (4)	0.75520 (14)	0.70016 (10)	0.0193 (3)	
H3O	0.695 (5)	0.855 (3)	0.7888 (16)	0.070 (9)*	

### Atomic displacement parameters (Å^2^)

**Table:**

	*U*^11^	*U*^22^	*U*^33^	*U*^12^	*U*^13^	*U*^23^
S1	0.01808 (17)	0.01776 (17)	0.01879 (19)	−0.00127 (16)	0.00143 (16)	−0.00022 (16)
O1	0.0388 (8)	0.0262 (7)	0.0175 (6)	−0.0044 (6)	0.0032 (5)	0.0031 (5)
O2	0.0208 (6)	0.0279 (7)	0.0271 (7)	0.0030 (6)	−0.0043 (5)	−0.0024 (6)
O3	0.0313 (7)	0.0287 (7)	0.0198 (6)	0.0057 (6)	−0.0071 (6)	−0.0065 (5)
N1	0.0228 (7)	0.0204 (7)	0.0157 (6)	−0.0026 (6)	0.0014 (6)	−0.0006 (5)
C1	0.0147 (8)	0.0162 (8)	0.0202 (8)	0.0017 (7)	−0.0005 (6)	−0.0009 (6)
C2	0.0217 (10)	0.0227 (9)	0.0179 (8)	0.0021 (7)	0.0002 (7)	0.0004 (7)
C3	0.0262 (9)	0.0293 (9)	0.0199 (8)	0.0010 (8)	−0.0050 (8)	−0.0042 (7)
C4	0.0220 (9)	0.0220 (8)	0.0311 (10)	−0.0046 (8)	−0.0031 (7)	−0.0033 (7)
C5	0.0208 (8)	0.0186 (8)	0.0253 (9)	−0.0002 (6)	0.0011 (7)	0.0026 (6)
C6	0.0184 (8)	0.0153 (7)	0.0191 (8)	0.0034 (7)	0.0006 (6)	0.0002 (6)
C7	0.0217 (9)	0.0159 (8)	0.0195 (9)	−0.0003 (7)	−0.0002 (7)	0.0017 (6)
C8	0.0229 (9)	0.0216 (8)	0.0190 (8)	0.0008 (7)	−0.0037 (7)	−0.0034 (6)
C9	0.0206 (9)	0.0201 (8)	0.0173 (8)	−0.0038 (7)	−0.0001 (7)	0.0029 (6)

### Geometric parameters (Å, °)

**Table:**

S1—N1	1.7079 (15)	C2—H2	0.9500
S1—C1	1.7385 (17)	C3—C4	1.403 (3)
O1—C7	1.249 (2)	C3—H3	0.9500
O2—C9	1.206 (2)	C4—C5	1.372 (2)
O3—C9	1.324 (2)	C4—H4	0.9500
O3—H3O	0.87 (3)	C5—C6	1.399 (2)
N1—C7	1.352 (2)	C5—H5	0.9500
N1—C8	1.447 (2)	C6—C7	1.454 (2)
C1—C6	1.395 (2)	C8—C9	1.511 (2)
C1—C2	1.395 (2)	C8—H8A	0.9900
C2—C3	1.378 (2)	C8—H8B	0.9900
			
N1—S1—C1	89.76 (8)	C4—C5—H5	120.7
C9—O3—H3O	112.0 (17)	C6—C5—H5	120.7
C7—N1—C8	121.51 (14)	C1—C6—C5	120.26 (15)
C7—N1—S1	116.59 (11)	C1—C6—C7	112.31 (15)
C8—N1—S1	121.90 (11)	C5—C6—C7	127.43 (15)
C6—C1—C2	121.30 (15)	O1—C7—N1	121.64 (15)
C6—C1—S1	111.95 (12)	O1—C7—C6	128.96 (17)
C2—C1—S1	126.75 (13)	N1—C7—C6	109.40 (14)
C3—C2—C1	117.61 (15)	N1—C8—C9	112.86 (14)
C3—C2—H2	121.2	N1—C8—H8A	109.0
C1—C2—H2	121.2	C9—C8—H8A	109.0
C2—C3—C4	121.52 (16)	N1—C8—H8B	109.0
C2—C3—H3	119.2	C9—C8—H8B	109.0
C4—C3—H3	119.2	H8A—C8—H8B	107.8
C5—C4—C3	120.75 (17)	O2—C9—O3	125.12 (17)
C5—C4—H4	119.6	O2—C9—C8	124.66 (16)
C3—C4—H4	119.6	O3—C9—C8	110.22 (15)
C4—C5—C6	118.55 (15)		
			
C1—S1—N1—C7	−0.51 (14)	C4—C5—C6—C7	178.74 (17)
C1—S1—N1—C8	−179.65 (14)	C8—N1—C7—O1	0.3 (3)
N1—S1—C1—C6	0.23 (13)	S1—N1—C7—O1	−178.81 (14)
N1—S1—C1—C2	179.76 (16)	C8—N1—C7—C6	179.77 (14)
C6—C1—C2—C3	0.8 (2)	S1—N1—C7—C6	0.63 (18)
S1—C1—C2—C3	−178.65 (14)	C1—C6—C7—O1	178.96 (17)
C1—C2—C3—C4	−0.6 (3)	C5—C6—C7—O1	0.1 (3)
C2—C3—C4—C5	0.0 (3)	C1—C6—C7—N1	−0.4 (2)
C3—C4—C5—C6	0.3 (3)	C5—C6—C7—N1	−179.27 (15)
C2—C1—C6—C5	−0.5 (2)	C7—N1—C8—C9	−80.1 (2)
S1—C1—C6—C5	179.01 (12)	S1—N1—C8—C9	99.03 (15)
C2—C1—C6—C7	−179.49 (15)	N1—C8—C9—O2	−8.2 (2)
S1—C1—C6—C7	0.06 (18)	N1—C8—C9—O3	172.04 (14)
C4—C5—C6—C1	0.0 (2)		

### Hydrogen-bond geometry (Å, °)

**Table:**

*D*—H···*A*	*D*—H	H···*A*	*D*···*A*	*D*—H···*A*
O3—H3O···O1^i^	0.86 (3)	1.72 (3)	2.581 (2)	173 (3)
C2—H2···O2^ii^	0.95	2.60	3.310 (2)	132
C8—H8A···O2^iii^	0.99	2.34	3.246 (2)	152

Symmetry codes: (i) −*x*+1, *y*+1/2, −*z*+3/2; (ii) *x*+1/2, −*y*+3/2, −*z*+1; (iii) *x*+1, *y*, *z*.
